# Exposure to β-hydroxybutyrate reduces the operating set point and increases excitability in hippocampal circuitry of healthy mice

**DOI:** 10.3389/fphar.2025.1557612

**Published:** 2025-10-29

**Authors:** Thais Tessari Zampieri, Guilherme Shigueto Vilar Higa, Fernando S. Borges, Felipe José Costa Viana, Emily Cruvinel, Lucas Eduardo Bentivoglio, Ademar Benevolo Lugao, Henning Ulrich, Luiz Roberto Britto, Kattesh V. Katti, Alton Michael Chesne, Roberto de Pasquale

**Affiliations:** ^1^ Laboratório de Neurofisiologia, Departamento de Fisiologia e Biofísica, Universidade de São Paulo, São Paulo, Brazil; ^2^ Laboratório de Neurociências, Departamento de Bioquímica, Instituto de Química (USP), São Paulo, Brazil; ^3^ Department of Physiology and Pharmacology, SUNY Downstate Health Sciences University, Brooklyn, NY, United States; ^4^ Physics Department at State University of Ponta Grossa, Ponta Grossa Paraná, Brazil; ^5^ Instituto de Pesquisas Energéticas e Nucleares IPEN-CNEN, São Paulo, Brazil; ^6^ Laboratório de Neurobiologia Celular, Departamento de Fisiologia e Biofísica, Universidade de São Paulo, São Paulo, Brazil; ^7^ Institute of Green Nanotechnology, Department of Radiology, School of Medicine, University of Missouri Columbia, Columbia, MO, United States; ^8^ Department of Research and Development, Tecton BG, INC, Alexandria, LA, United States

**Keywords:** β-hydroxybutyrate (BHB), hippocampus, electrophysiology, long-term potentiation (LTP), brain slice, ketone, niacin

## Abstract

The ketogenic diet is a therapeutic strategy applied to reduce brain hyperexcitability in conditions such as epilepsy, Parkinson’s and Alzheimer’s disease, migraines, and autism. This diet reduces circulating glucose levels and increases ketone bodies, with β-hydroxybutyrate (BHB) being one of the leading promoters of the beneficial effects. BHB was previously reported as a mediator of cognitive restoration and memory formation. Herein, we investigate the effect of exogenous BHB on hippocampal neuronal excitability and synaptic plasticity mechanisms, regardless of the pathological or neurodegenerative conditions. Electrophysiological experiments were conducted to explore both passive and active neuronal properties, including action potential firing and spontaneous and evoked postsynaptic responses. Electrical stimulation along the CA3-CA1 pathway enabled the assessment of both short- and long-term synaptic plasticity, as well as the mechanisms mediated by AMPA and NMDA receptors. Experiments were conducted in hippocampal slices treated with 3-β-hydroxybutyrate glycerides (DHB) and niacin (HCAR2 agonist). Although DHB incubation did not alter passive membrane properties, it significantly increased neuronal excitability, reflected in an elevated firing rate upon depolarizing stimulation and enhanced spontaneous excitatory postsynaptic currents in CA1 pyramidal neurons, which were dependent on synaptic inputs. DHB treatment led to a reduction in long-term potentiation (LTP) in CA1 neurons, suggesting a metaplastic effect independent of NMDA receptor activation. Importantly, these DHB-induced neuronal alterations were found to be independent of HCAR2 receptor activation, supporting the involvement of distinct intracellular pathways and long-term modulatory mechanisms. Our findings indicate that DHB exerts a modulatory effect on hippocampal neural activity by enhancing excitability and concurrently promoting a compensatory reduction in LTP, suggesting a homeostatic balancing mechanism.

## 1 Introduction

For more than a century, the ketogenic diet (KD), a low-carbohydrate, adequate protein, and high-fat diet, has been indicated and used in association with the treatment of a wide variety of neurological disorders such as migraine, epilepsy, multiple sclerosis, Parkinson’s disease and Alzheimer’s disease (Wheless, 2008; Price and Ruppar, 2023; Al-kuraishy et al., 2024). Indeed, KD is routinely applied in association with pharmacological treatment in patients with epilepsy (Zarnowska, 2020; Mishra et al., 2024), a group of brain disorders characterized by overexcitability in distinct brain regions (Sepkuty et al., 2002). Clinical studies indicate that KD improves control of epileptic seizures (Freeman and Kossoff, 2010; Rogawski et al., 2016) and diminishes neuronal hyperexcitability manifested in other neuronal disorders (Simeone and Simeone, 2024; Hertz et al., 2015; Gross et al., 2019; Cheng et al., 2020; Fila et al., 2023; Iyer et al., 2024).

The KD mimics starvation by promoting fat metabolism as the primary energy source, reducing circulating glucose levels, and increasing ketone bodies (KB) (Freeman, 2006; Masino and Rho, 2019). In this way, not only KD but also intermittent fasting becomes an alternative tool to guarantee higher levels of circulating BHB, the main KB that promotes beneficial effects (Kim et al., 2015; Rho et al., 2019; Mohamed et al., 2024). Although KB has a positive impact on all systemic components of metabolism, such as weight loss, glycemic control, lower blood pressure, improved lipid profiles, improved cardiac function, and enhanced vascular function (Faria-Costa et al., 2024; Soni et al., 2024), a growing number of clinical studies have reported the impact of fasting and caloric restriction on neurodegenerative diseases (Hansen et al., 2024).

KB generated by fatty acid oxidation serves as alternative metabolites for aerobic energy production. In the brain, BHB is metabolically converted into acetyl-CoA, which enters the tricarboxylic acid cycle. NADH and FADH2 facilitate ATP synthesis via oxidative phosphorylation in the inner mitochondrial membrane (Veech et al., 2001; Vidali et al., 2015). Experiments demonstrated that the ketotic brain metabolizes less glucose and more acetate, producing a large amount of acetyl-CoA and consequently altering the equilibrium of the aspartate aminotransferase reaction. This process results in an enhanced synthesis of glutamine and GABA (Yudkoff et al., 2005).

The mechanisms of KB include improvement of mitochondrial function and intestinal microbiota. In the brain, KB decreases neuronal excitability by modulating neurotransmitters, ion channels, and receptors (Ma et al., 2007; Rogawski et al., 2016; Mishra et al., 2024). KB promotes inhibition of glutamate release by competing with Cl^−^ at the site of VGLUT allosteric regulation. Acetoacetate reduced quantal size at hippocampal synapses and suppressed glutamate release (Juge et al., 2010). Also, acetoacetate and BHB reduce the firing rate of GABAergic neurons in the substantia nigra pars reticulata, a putative subcortical seizure gate (Ma et al., 2007).

Likewise, KD and BHB can fully restore long-term potentiation (LTP) maintenance in the epileptic mouse model (Kim et al., 2015). Similarly, an AD model (APP/PS1) demonstrated that the KD can increase neuronal plasticity markers (ERK and CREB). Therefore, it appears that BHB would reestablish LTP function in the hippocampus under pathological conditions (Di Lucente et al., 2024) and increase neuronal plasticity markers, such as GluN2A, Glu-A1, Snap25, and PSD-95 (Paidi et al., 2023; Paidi and Pahan, 2024). Interestingly, cognitive improvements associated with physical exercises correlate with an increased BHB production. In this circumstance, BHB promotes increased BDNF synthesis by targeting the HDAC2 and HDAC3 receptors (Sleiman et al., 2016).

Given the effects of the KD and its product, the BHB, we investigate the specific action of BHB on neuronal excitability and synaptic plasticity mechanisms, regardless of pathological or neurodegenerative conditions. Thus, this study sheds light on understanding the basal function of BHB under physiological conditions.

## 2 Materials and methods

### 2.1 Animal model

All procedures used in this study were approved by the Ethics Committee for Animal Use (CEUA) from the Biomedical Sciences Institute of São Paulo University (Protocol 4515150223). C57BL-6 male mice were used at the age of postnatal day 22-28 (P22-28). The animals were kept in their respective litters in the animal facility of the Department of Physiology and Biophysics at ICB/USP under standard conditions: 23 °C ± 2 °C, 12 h light/dark cycle, light-dark cycle of 12: 12 h (lights on 6 h a.m.), food and water *ad libitum*, and lighting ∼200 lx.

### 2.2 Brain slices preparation

Mice were anesthetized using isoflurane inhalation (Neo Quimica^®^), decapitated and the brain was quickly removed and placed in cooled (<4 °C) oxygenated (5% CO_2_- 95% O_2_) dissection buffer containing the following (in mM): 92 NaCl, 30 NaHCO_3_, 25 D-glucose, 20 HEPES, 10 MgSO_4_, 3 Na-pyruvate, 2.5 KCl, 1.25 NaH_2_PO_4_ and 0.5 CaCl_2_. Once in solution, horizontal slices of the hippocampus (300–350 µm) were obtained using a vibratome (VT 1200-S, Leica Biosystems; Heidelberger Str. 17–19, 69226 Nussloch, Baden-Württemberg, Germany). Slices were transferred immediately after cutting to a chamber containing artificial cerebrospinal fluid (aCSF) freshly prepared, which contained (in mM): 124 NaCl, 24 NaHCO_3_, 12.5 D-Glucose, 5 HEPES, 2.5 KCl, 2 MgSO_4_, 2 CaCl_2_, and 1.5 NaH_2_PO_4_ in the presence of carbogen (5% CO_2_- 95% O_2_) at pH 7.3-7.4. Slices were kept oxygenated at room temperature (20 °C–25 °C) for at least 1 h before proceeding with electrophysiological recordings.

### 2.3 Pharmacology

We used 3-β-hydroxybutyrate glycerides (2 mM, DHB; Tecton), a combination molecule of ketone and glycerin, and glycerin (0.2 mM; ACS Cientifica: Cat. No. R08071000) as the vehicle from the Control group in all experiments. Both were prepared immediately before experiments by dilution in artificial cerebrospinal fluid (aCSF) to reach their final concentration. DHB at a 2 mM dose was chosen by the fact that this concentration is compatible with the plasma values of patients on a ketogenic diet and outside the harmful values of ketoacidosis (>3 mM) (Lopaschuk and Dyck, 2023).

The effects of DHB were studied using two strategies: 1) slices were incubated for 2 h before recording; in this case, the control group was incubated with glycerin; 2) a baseline recording was carried out, and DHB was added to the bath to evaluate its acute effect.

To evaluate intrinsic excitability, extra synaptic blockers were added to aCSF before current steps: selective and non-competitive NMDAr antagonist (50 μm, MK-801; Tocris, Cat. No. 0924); AMPA/Kainate Glutamate receptor antagonist (10 μm, DNQX; Tocris: Cat. No. 0189); GABA_A_ receptor antagonist (50 μM, PTX; Tocris: Cat. No. 1128).

To assess AMPAr currents, dizocilpine (50 μm, MK-801; Tocris, Cat. No. 0924), a selective and non-competitive NMDAr antagonist, was added to aCSF. NMDAr-mediated responses were performed with aCSF containing 6,7-dinitroquinoxaline-2,3-dione (10 μm, DNQX; Tocris: Cat. No. 0189) and Picrotoxin (50 μM, PTX; Tocris: Cat. No. 1128), an AMPA/Kainate Glutamate receptor antagonist and a GABA_A_ receptor antagonist, respectively.

To verify whether the response to DHB was mediated via its Hydroxycarboxylic Acid Receptor 2 (HCAR2), we used the non-selective agonist Niacin (Nicotinic acid, Sigma-Aldrich: Cat. No. N4126), which was prepared immediately before the experiments by dilution in aCSF to reach a final concentration of 100 μM. A 100 μM concentration of niacin was selected because it is sufficient to bind HCAR2 and promote close to maximal activation of its functional response (Wise et al., 2003). Two strategies were adopted: 1) slices were incubated for 2 h before recording; 2) a baseline recording was carried out, and Niacin was added to the bath to evaluate its acute effect.

### 2.4 Electrophysiological recordings

Hippocampal slices were placed in a chamber (submersion-type) upon a differential interference contrast-equipped Nikon Eclipse E600FN microscope stage, and the temperature was maintained constant and continuously monitored inside the chamber at 30 °C throughout the experiment using a solution in-line heater (Warner Intrsuments^®^; Cat. No. 64-0103). The chamber was kept in constant perfusion with oxygenated aCSF. Micropipettes were fabricated from borosilicate glass (Harvard Apparatus) with 3–4 MΩ input resistance, filled with intracellular solution containing the following (in mM): 117 K-gluconate, 13 KCl, 10 HEPES, 2 Na_2_ATP, 1 MgCl_2_, 0.4 Na_3_GTP, 0.1 EGTA and 0.07 CaCl_2_, with pH 7.3 and osmolality 290 ± 10 mOsm. CA1 pyramidal neurons were identified and patched, and whole-cell recordings were obtained using a Multiclamp 700B amplifier and pClamp 10 software (Molecular Devices). Only regular spiking pyramidal-shaped cells were included in the data. Cells with any of the following were excluded from experiments: <-55 mV resting membrane potential, failing to display action potentials at the beginning and end of the recording, >20 MΩ access resistance, <120 MΩ input resistance, and >1000 MΩ input resistance.

### 2.5 Neuronal firing

In experiments where the action potential (AP) firing was analyzed, electrical features under current injection in pyramidal CA1 neurons were extracted using the Electrophys Feature Extraction Library (eFEL) [https://github.com/BlueBrain/eFEL]. Slices were pre-incubated with glycerin (control), DHB, niacin, or DHB + niacin for 2 h before recordings. Neurons were stimulated with 1000 ms current steps (−110, −90, −70, −50, −30, −10, 10, 30, 50, 70, 90, 110, 130, 150, and 170 pA). Resting membrane potential and capacitance were determined after signal stabilization. The current-voltage (I-V) relationship was plotted, and input resistance was calculated for each cell. The −110 pA pulse was used to calculate the capacitance, defined as the ratio of time constant and access resistance, and SAG current, defined as the relative decrease from the maximum hyperpolarization to the steady-state voltage. The rheobase was defined as the minimum current step that elicits AP firing. The first depolarizing current step was used to analyze the AP peak amplitude, after-hyperpolarization (AHP) amplitude, and time to the first spike. Fire rate was calculated by counting spikes for each current level. To evaluate intrinsic excitability, Control and DHB slices were prepared, and current steps were recorded in the absence of synaptic inputs (AMPA, NMDA and GABAa blockers).

### 2.6 Spontaneous excitatory and inhibitory postsynaptic currents

The recording of spontaneous Excitatory Postsynaptic Currents (sEPSC) responses was performed using K-gluconate intracellular solution (as described in 2.4 item) in voltage-clamp mode with a holding potential of −70 mV. Slices were pre-incubated with glycerin (control), DHB, or niacin for 2 h before recordings. The basal activity of spontaneous postsynaptic currents was recorded for 20 min from Control, DHB, and niacin slices. To quantify spontaneous Inhibitory Postsynaptic Currents (sIPSC), slices were transferred to a chamber containing oxygenated aCSF with 20 µM DNQX and 50 µM MK-801. To record sIPSC, micropipettes (3–4 MΩ) were filled with intracellular solution containing the following (in mM): 130 CsCl, 10 HEPES, 10 TEA, 6 QX-314, 5 EGTA, 5 Na_2_-creatine phosphate, 4 MgATP, and 0.5 Na_2_GTP, with pH 7.3 and osmolality 290 ± 10 mOsm. Recording of sIPSC responses was performed in voltage-clamp mode with a holding potential of −70 mV.

The recording of miniature Excitatory Postsynaptic Currents (mEPSC) was performed as described elsewhere (Carlos-Lima et al., 2023). Slices were pre-incubated with glycerin (control) or DHB 2 h before recordings. Cs-Cl intracellular solution, composed by (in mM): 30 CsCl, 10 HEPES, 5 EGTA, 5 Na-Phosphocreatine, 4 MgATP, 0.5 NaGTP, 10 TEA, and 5 QX-314. (pH 7.3 and the osmolarity 290 mOsm) was used to record CA1 pyramidal neurons under the effects of DHB or glycerin (control group) in voltage-clamp mode with a holding potential of −70 mV. The mEPSC were isolated by applying the GABA_A_ receptor antagonist Picrotoxin (50 μM; Tocris: Cat. No. 1128) and TTX (0.5 μM; Tocris: Cat. No. 1078).

Spontaneous and miniature excitatory postsynaptic currents were analyzed using the Electrophys Feature Extraction Library (eFEL) [https://github.com/BlueBrain/eFEL]. All events were detected with a threshold (minimum values of amplitude, width, and prominence) and re-examined visually for data acceptance. The analyzed parameters included inter-event interval (ms), peak amplitude (pA), charge transfer (fC), rise time (ms), decay time (ms), width (ms), frequency (Hz) and synaptic current densities were calculated by normalizing total currents to cell membrane capacitance as previous described (Netsyk et al., 2020; Netsyk et al., 2025). Data were presented as cumulative probability, and 50% probability (P50) was considered to the comparative analyses.

### 2.7 Electrical stimulation and short-term plasticity

A concentric bipolar electrode (1.25 mm diameter) was placed in contact with Schaffer collateral. Recording from whole-cell pyramidal CA1 neurons was performed during electrical stimulation at the stratum radiatum of CA1 proximal to CA3 (evoked Postsynaptic Potential by Schaffer collateral stimulation - WPS Stimulus Isolator; Model A365) in current-clamp mode with a holding current of zero. Slices were pre-incubated with glycerin (control) or DHB 2 h before recordings. Input-output synaptic response curves were studied by first establishing the minimal stimulation intensity (MSI). The MSI was determined by gradually increasing the stimulation intensity in 5 µA increments until an evoked excitatory postsynaptic potential (eEPSPs) was distinguishable from noise. Subsequently, the eEPSPs data points for the eEPSPs of the curve were assessed by further increasing the stimulation intensity; these values were normalized relative to the MSI (1.0, 1.5, 2.0, 2.5, 3.0, 3.5, and 4.0). To estimate the pre- or postsynaptic nature of the modulatory effects on synaptic transmission and plasticity, we analyzed changes in the paired-pulse ratio (PPR). We delivered a paired-pulse protocol consisting of 5 sets of 2 pulses at different time intervals (200, 150, 100, 50, and 25 ms). The eEPSP amplitudes were averaged to obtain a mean value for each set. The PPR was then calculated as the ratio between the second and the first eEPSP.

### 2.8 Electrical stimulation and long-term plasticity

A concentric bipolar electrode (1.25 mm diameter) was placed in the *stratum radiatum* of CA1 (proximal to CA3) to stimulate the Schaffer collateral. The recording electrode was placed in the stratum radiatum of CA1, proximal to the *subiculum*, and the electrode measured the extracellular field excitatory postsynaptic potential (fEPSP) when the stimulation electrode triggered a pulse of current. To record fEPSP, micropipettes (1–2 MΩ) were filled with aCSF. Slices were pre-incubated with glycerin (control) or DHB 2 h before recordings. The experimental design started with an evoked fEPSP baseline (stimulation duration of 0.2 ms, 0.3 Hz) record that was stable for at least 20 min. To evaluate synaptic plasticity at CA1, we used high-frequency stimulation (HFS), consisting of 1 second train of 100 Hz (pulse duration 0.2 ms) repeated 4 times at 20-s intervals. The stimulation intensity was previously determined by 30%–40% of slope saturation. HFS was applied after a stable 20-min baseline recording. After the high-frequency stimulation, synaptic responses were further recorded for 60 min. Changes in fEPSP slope induced by HFS were quantified by calculating the normalized average of the last 10 min from baseline and comparing this value with the normalized slope average of each minute from the record for posterior comparison between control and DHB groups.

### 2.9 Mechanisms of evoked postsynaptic responses

Upon patching, a protocol of depolarizing current steps was applied to check for regular spiking. Slices were pre-incubated with glycerin (control) or DHB for 2 h before recordings. The isolation of NMDAr-evoked postsynaptic currents (eEPSC) was achieved by applying specific blockers. With a concentric bipolar electrode (1.25 mm diameter) placed in contact with Schaffer collateral, it was triggered pulses of current (pA) gradually increased (input: 1.0, 1.25, 1.50, 1.75, 2.0, and 2.25) to evaluate eEPSC (output) in pyramidal neurons of CA1. The stimulation intensities were normalized to the minimal response. To access the responses related to the activation of AMPA receptors, 10 min before recording, slices remained in aCSF containing MK-801 (50 µM) a NMDA receptor antagonist. Recording of AMPA-mediated eEPSC was performed in voltage-clamp mode with a holding potential of −56 mV. Recording of NMDAr activation was performed with ionotropic glutamatergic antagonist DNQX (10 µM) in the modified aCSF (Mg^+2^ free) to block the currents through AMPAr and Picrotoxin (50 µM) to block the currents through GABAergic ionotropic receptors. Recording of NMDAr-mediated eEPSC was performed in voltage-clamp mode with a holding potential of −70 mV.

For analysis of the effect of DHB and niacin on synaptic activity and its underlying mechanisms, we evaluate eEPSP in CA1 pyramidal cells by Schaffer collateral. After recording the stable baseline for at least 5 min (perfused with normal aCSF), DHB, niacin, or DHB plus niacin were applied in the bath to their final concentrations (2 mM, 100 μM, respectively), and the recording continued for 20 min.

### 2.10 Statistical methods

For all data, normality was tested using the Shapiro-Wilk test. In experiments where data were normally distributed, statistical analysis was performed using parametric tests. When the experimental groups were compared, the unpaired t-test was used. In experiments where the data were not normally distributed and the mean differences between groups were split by one variable, statistical analysis was performed using non-parametric tests. When the experimental groups were two independent samples, the Mann-Whitney U test was used. The statistical analysis of the differences in firing rate, eEPSP, eEPSC, and Paired-Pulse between the two groups was carried out using a Two-Way repeated measures ANOVA using the Bonferroni post test. Active and Passive membrane Properties were statistically analyzed by Two-Way ANOVA using the Bonferroni post test. All experimental groups were considered significantly different for p-values lower than 0.05. Data were presented as mean ± SEM (standard error of the mean) and violin plot indicate the median along with the interquartile range (25th and 75th percentiles). Statistical analysis was performed with GraphPad (Prism version 10.5.0).

## 3 Results

### 3.1 Hippocampal excitability is increased by incubation with 3-β-hydroxybutyrate glycerides

**FIGURE 1:**
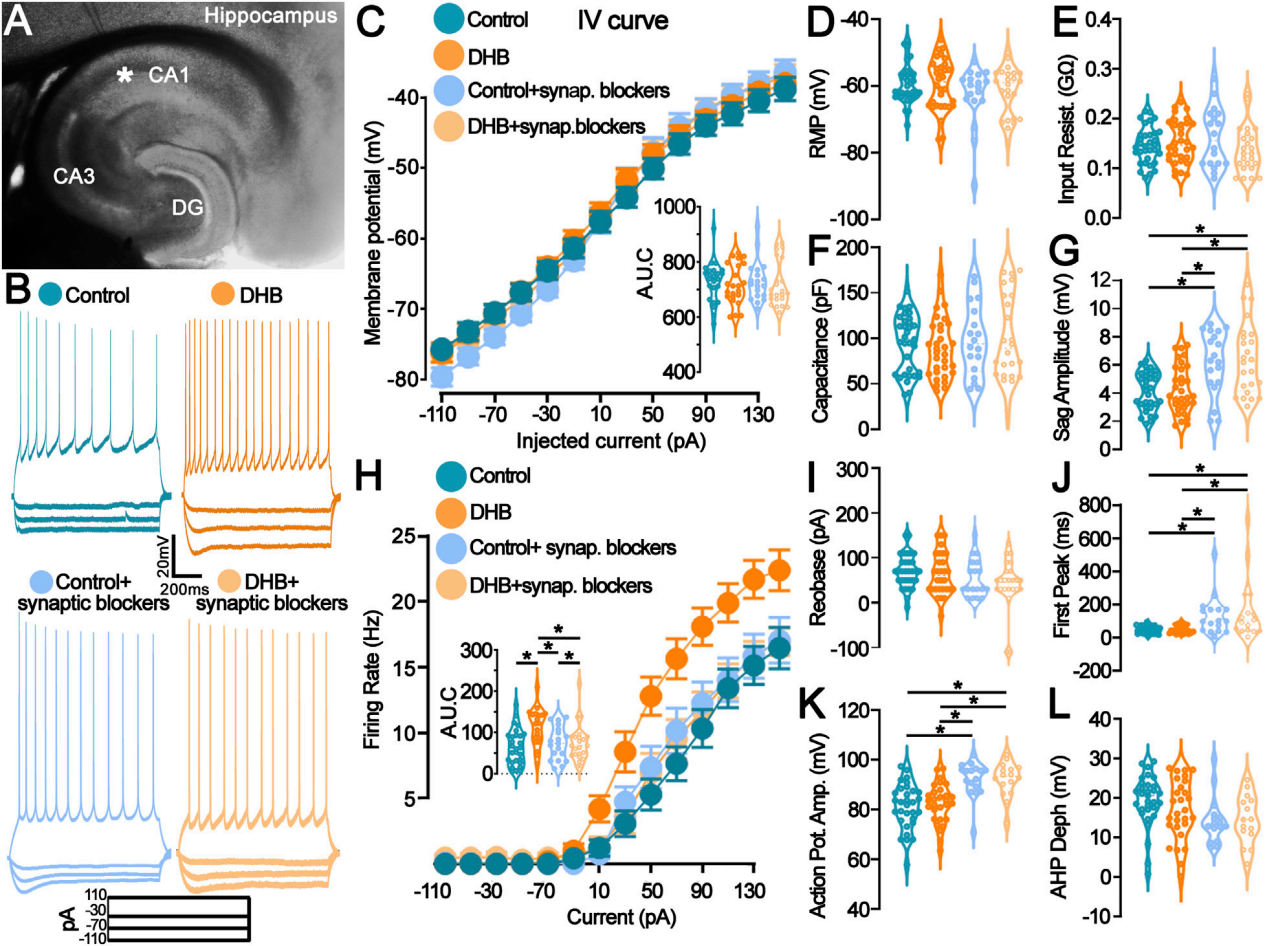
Incubation with 3- β-hydroxybutyrate glycerides (DHB) increases the pyramidal neuron firing rate in a synaptic receptor-dependent manner. **(A)** Representative photomicrograph shows the location of the recording electrode in the mouse hippocampal slice: pyramidal CA1 neurons were studied. **(B)** Example traces from pyramidal neurons are shown: Vm (top) in response to the step current injections (bottom) of all four groups. **(C)** Current-Voltage plot (I-V relationship) for the whole-cell current measurements showed no difference (Control: 28 cells, 11 animals; DHB: 20 cells, 12 animals; Control+ synaptic blockers: 19 cells, 6 animals; DHB+ synaptic blockers: 24 cells, 6 animals; Three-Way ANOVA, Control vs. DHB p = 0.4867, Normal vs. Blockers p = 0.9274, Injected current x Control vs. DHB x Normal vs. Blockers p = 0.0433); Intergraph: Area under de curve (AUC) (Two-Way ANOVA, DHB p = 0.4905, Blockers p = 0.9277, Interaction p = 0.9978). **(D–G)** Graphs representing the passive membrane properties (Control: 33 cells, 16 animals; DHB: 32 cells, 13 animals; Control + blockers: 19 cells, 6 animals; DHB + blockers: 24 cells, 6 animals). **(D)** Resting membrane potential (RPM, Control: -63.4 ± 1.1; DHB: -63.5 ± 2.2; Control + blockers: -62.8 ± 2.1; DHB + blockers: -61.6 ± 1.5; Two-Way ANOVA, DHB p = 0.7395, Blockers p = 0.0661, Interaction p = 0.6256); **(E)** Input resistance (Control: 0.1820 ± 0.0099; DHB: 0.1651 ± 0.0121; Control + blockers: 0.1615 ± 0.0141; DHB + blockers: 0.1375 ± 0.0095; Two-Way ANOVA, DHB p = 0.3681, Blockers p = 0.9428, Interaction p = 0.0905); **(F)** Capacitance (Control: 94.4 ± 5.5; DHB: 89.3 ± 5.7; Control + blockers: 98.3 ± 9.2; DHB + blockers: 101.0 ± 9.6; Two-Way ANOVA, DHB p = 0.8739, Blockers p = 0.2958, Interaction p = 0.6015); **(G)** Sag amplitude (Control: 4.15 ± 0.24; DHB: 4.28 ± 0.30; Control + blockers: 6.18 ± 0.52; DHB + blockers: 6.45 ± 0.51; Two-Way ANOVA, DHB p = 0.6009, Blockers p < 0.0001, Interaction p = 0.8536, horizontal bars indicate p < 0.05 in multiple comparisons). **(H)** Current versus firing rate relationships (FI curves), averaged over pyramidal cells recorded in each group (Control: 28 cells, 11 animals; DHB: 20 cells, 12 animals; Control+ synaptic blockers: 19 cells, 6 animals; DHB+ synaptic blockers: 23 cells, 6 animals; 3Way ANOVA, Control vs. DHB p = 0.0102, Normal vs. synaptic blockers p = 0.1044, Injected current x Control vs. DHB x Normal vs. Blockers p > 0.0001); Intergraph: Area under de curve (AUC) (Two-Way ANOVA, DHB p = 0.0099, Blockers p = 0.1052, Interaction p = 0.0105, horizontal bars indicate p < 0.05 in multiple comparisons). **(I–L)** Graphs representing the active membrane properties (Control: 33 cells, 16 animals; DHB: 32 cells, 13 animals; Control + synaptic blockers: 19 cells, 6 animals; DHB + synaptic blockers: 24 cells, 6 animals). **(I)** Rheobase (Control: 70.65 ± 6.65; DHB: 64.67 ± 8.57; Control + blockers: 54.44 ± 9.09; DHB + blockers: 42.50 ± 11.41; Two-Way ANOVA, DHB p = 0.3338, Blockers p = 0402, Interaction p = 0.7471); **(J)** Time to first spike (Control: 47.18 ± 3.90; DHB: 50.61 ± 4.40; Control + blockers: 131.9 ± 28.47; DHB + blockers: 165.2 ± 47.95; Two-Way ANOVA, DHB p = 0.3790, Blockers p < 0.0001, Interaction p = 0.4744, horizontal bars indicate p < 0.05 in multiple comparisons); **(K)** Action potential amplitude (Control: 81.24 ± 1.63; DHB: 82.41 ± 1.47; Control + blockers: 92.27 ± 1.71; DHB + blockers: 92.12 ± 1.73; Two-Way ANOVA, DHB p = 0.7717, Blockers p < 0.0001, Interaction p = 0.7048, horizontal bars indicate p < 0.05 in multiple comparisons); **(L)** After hyperpolarization depth (Control: 19.33 ± 1.17; DHB: 16.28 ± 1.53; Control + blockers: 14.29 ± 1.47; DHB + blockers: 14.49 ± 1.59; Two-Way ANOVA, DHB p = 0.4619, Blockers p = 0.0144, Interaction p = 0.3852).

First, we performed a whole-cell patch-clamp of neurons located in the CA1 region of the hippocampus to investigate the effects of 3-β-hydroxybutyrate glycerides (DHB) on the biophysical properties of pyramidal neurons (Figure 1). Incubation with DHB significantly increased the firing rate of CA1 pyramidal neurons without altering other intrinsic biophysical properties. However, these changes seem to be synapse-dependent.

Figure 1A displays a representative photomicrograph indicating the placement of the recording electrode within the hippocampal slice, targeting CA1 pyramidal neurons. Representative voltage traces in response to step current injections are shown in Figure 1B. Whole-cell current-voltage (I–V) relationships (Figure 1C) did not differ significantly between groups (Control vs. DHB: p = 0.4867), indicating that DHB does not affect passive membrane conductance. AUC analysis corroborated the absence of significant changes (DHB: p = 0.4905; Interaction: p = 0.9978). In contrast, the input-output relationship, assessed through frequency-current (FI) curves (Figure 1H), revealed a significant enhancement in neuronal excitability following DHB treatment compared to control (3-way ANOVA, Control vs. DHB: p = 0.0102). Interestingly, this effect was abolished in the presence of synaptic blockers (Control vs. Blockers: p = 0.1044), suggesting that the DHB-induced increase in firing rate is contingent upon synaptic signaling. Quantification of the area under the curve (AUC) further supported this observation (Two-Way ANOVA, DHB: p = 0.0099; Interaction: p = 0.0105).

Passive membrane properties, including resting membrane potential, input resistance, membrane capacitance, and sag amplitude, are summarized in Figures 1D–G. No significant differences were observed between control and DHB-treated neurons across these parameters (all p > 0.05). However, the presence of blockers significantly increased sag amplitude (Blockers: p < 0.0001), suggesting modulation of hyperpolarization-activated conductances by synaptic inputs. Active membrane properties, including rheobase, time to first spike, action potential amplitude, and afterhyperpolarization depth (Figures 1I–L), remained unaffected by DHB treatment (all p > 0.05). Nevertheless, synaptic blockers significantly altered several parameters: time to first spike (p < 0.0001), action potential amplitude (p < 0.0001), and afterhyperpolarization depth (p = 0.0144), indicating that synaptic inputs contribute to shaping the active electrophysiological profile of CA1 pyramidal neurons independently of DHB.

These data collectively demonstrate that, although DHB does not affect the membrane properties, it can enhance neuronal responsiveness by increasing the frequency of action potential firing in response to depolarization when synaptic inputs are preserved.

### 3.2 Excitatory spontaneous currents, but not inhibitory, show increased activity after incubation with 3-β-hydroxybutyrate glycerides

**FIGURE 2:**
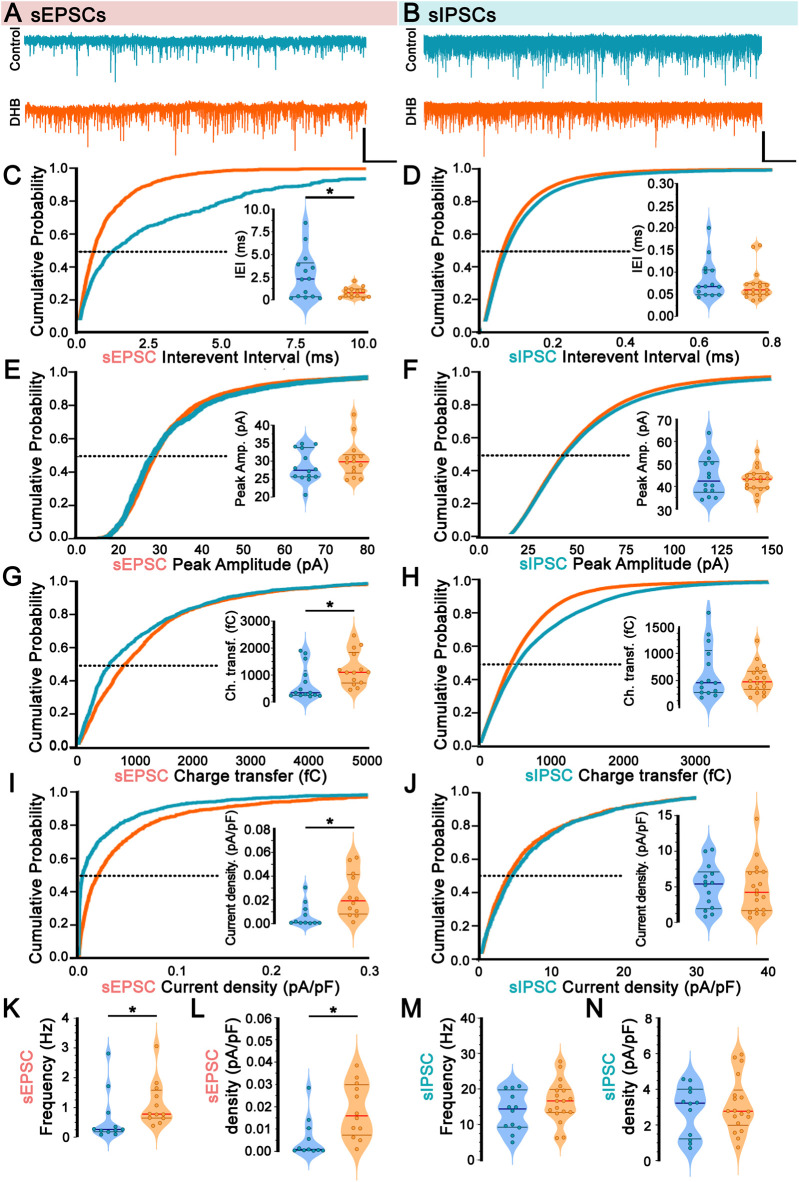
Incubation with 3-β-hydroxybutyrate glycerides (DHB) upregulates spontaneous excitation in CA1 pyramidal neurons. **(A,B)** Representative currents and averaged currents from individual cells are shown for sEPSC **(A)** (Control: 14 cells, 7 animals *vs*. DHB: 13 cells, 7 animals) and sIPSC **(B)** (Control: 14 cells, 7 animals *vs*. DHB: 18 cells, 7 animals). **(C)** Cumulative probability distribution of spontaneous Excitatory Postsynaptic Currents (sEPSC) interevent interval; inset: average sEPSC interevent interval at P50 (Control: 2.64 ± 0.67 *vs*. DHB: 0.89 ± 0.15; unpaired t-test, p = 0.0217). **(D)** Cumulative probability distribution of spontaneous Inhibitory Postsynaptic Currents (sIPSC) interevent interval: inset: average sIPSC interevent interval at P50 (Control: 0.084 ± 0.0.012 *vs*. DHB: 0.071 ± 0.008; Mann-Whitney U test, p = 0.4815). **(E)** Cumulative probability distribution of sEPSC peak amplitude; inset: average sEPSC peak amplitude at P50 (Control: 28.54 ± 1.18 *vs*. DHB: 30.50 ± 1.38; Mann-Whitney U test, p = 0.4544). **(F)** Cumulative probability distribution of sIPSC peak amplitude; inset: average sIPSC peak amplitude at P50 (Control: 44.54 ± 2.37 *vs*. DHB: 43.03 ± 1.24; unpaired t-test, p = 0.5507). **(G)** Cumulative probability distribution of sEPSC area; inset: average sEPSC area at P50 (Control: 694.5 ± 167.8 *vs*. DHB: 1231.0 ± 168.6; Mann-Whitney U test, p = 0.0091). **(H)** Cumulative probability distribution of sIPSC area; inset: average sIPSC area at P50 (Control: 6,650 ± 1319 *vs*. DHB: 5,355 ± 626; Mann-Whitney U test, p = 0.8662). **(I)** Cumulative probability distribution of sEPSC current density; inset: average sEPSC current density at P50 (Control: 0.0067 ± 0.0030 *vs*. DHB: 0.0240 ± 0.0054; Mann-Whitney U test, p = 0.0055). **(J)** Cumulative probability distribution of sIPSC current density; inset: average sIPSC current density at P50 (Control: 5.05 ± 0.83 *vs*. DHB: 4.81 ± 0.85; unpaired t-test, p = 0.8453). **(K)** sEPSC frequency (Control: 0.646 ± 0.260 *vs*. DHB: 1.123 ± 0.219; Mann-Whitney U test, p = 0.0188). **(L)** Total sEPSC density (Control: 0.0057 ± 0.0027 *vs*. DHB: 0.0183 ± 0.0035; Mann-Whitney U test, p = 0.0044). **(M)** sIPSC frequency (Control: 13.66 ± 1.59 *vs*. DHB: 16.56 ± 1.38; unpaired t-test, p = 0.1858). **(N)** Total sIPSC density (Control: 2.83 ± 0.41 *vs*. DHB: 3.12 ± 0.37; unpaired t-test, p = 0.6009).

The functionality of neural networks depends on the relationship between excitation and inhibition, which is defined as the E/I ratio. To assess the long-term effects of DHB on the E/I ratio, a set of whole-cell electrophysiological recordings was conducted on pyramidal CA1 neurons by comparing cells treated for 2 hours with DHB with control neurons. These recordings aimed to investigate the network activity by measuring spontaneous excitatory postsynaptic currents (sEPSCs) and spontaneous inhibitory postsynaptic currents (sIPSCs) (Figure 2). Representative sEPSC from individual cells are shown in Figure 2A, and Representative sIPSC from individual cells are shown in Figure 2B.

Analysis of inter-sEPSC intervals revealed a significant difference following exposure to DHB (control: 2.64 ± 0.67 *vs*. DHB: 0.89 ± 0.15; p = 0.02) (Figure 2C), combined by an increase in sEPSC frequency (Control: 0.646 ± 0.260 *vs*. DHB: 1.123 ± 0.219; Mann-Whitney U test, p = 0.0188) (Figure 2K). Although no significant differences were found in peak amplitude (control: 28.54 ± 1.18 *vs*. DHB: 30.50 ± 1.38; p = 0.45) (Figure 2E), rise time (control: 2.03 ± 0.12 *vs*. DHB: 2.07 ± 0.19; p = 0.88), decay time (control: 5.23 ± 0.23 *vs*. DHB: 5.72 ± 0.48; p = 0.31), and width parameters (control: 7.39 ± 0.35 *vs*. DHB: 8.02 ± 0.62; p = 0.38), an increase in the sEPSC charge transfer was observed in CA1 pyramidal neurons pre-treated with DHB (control: 694.5 ± 167.8 *vs*. DHB: 1231.0 ± 168.6; p = 0.009) (Figure 2G). To ensure comparability across cells of varying sizes, we adjusted the total synaptic current load (calculated as charge per event multiplied by event frequency) by normalizing it to membrane capacitance, the cumulative probability distribution of sEPSC current density (Figure 2I) was also higher in DHB group, together with sEPSC frequency (Figure 2K) and sEPSC density (Figure 2L), it suggests that the net excitatory drive per membrane area is increased after DHB exposure.

Despite the changes in sEPSC, spontaneous inhibitory activity remained unaltered following DHB application. For all parameters analyzed related to spontaneous inhibitory postsynaptic currents (sIPSC), no significant differences were observed: interevent interval (control: 0.08 ± 0.001 vs. DHB: 0.07 ± 0.01; p = 0.48) (Figure 2D); peak amplitude (control: 44.54 ± 2.37 vs. DHB: 43.03 ± 1.24; p = 0.55) (Figure 2F); area (control: 6650 ± 1319 vs. DHB: 5355 ± 626; p = 0.87) (Figure 2H); rise time (control: 1.04 ± 0.1 vs. DHB: 1.04 ± 0.06; p = 0.95) decay time (control: 4.98 ± 0.25 vs. DHB: 4.97 ± 0.19; p = 0.98) and width (control: 6.37 ± 0.38 vs. DHB: 6.33 ± 0.25; p = 0.93); sIPSC current density (control: 5.05 ± 0.83 v.s. DHB: 4.81 ± 0.85; p = 0.8453) (Figure 2J); sIPSC frequency (control: 13.66 ± 1.59 vs. DHB: 16.56 ± 1.38; p = 0.1858) (Figure 2M); total sIPSC density (control: 2.83 ± 0.41 vs. DHB: 3.12 ± 0.37; p = 0.6009) (Figure 2N).

**FIGURE 3:**
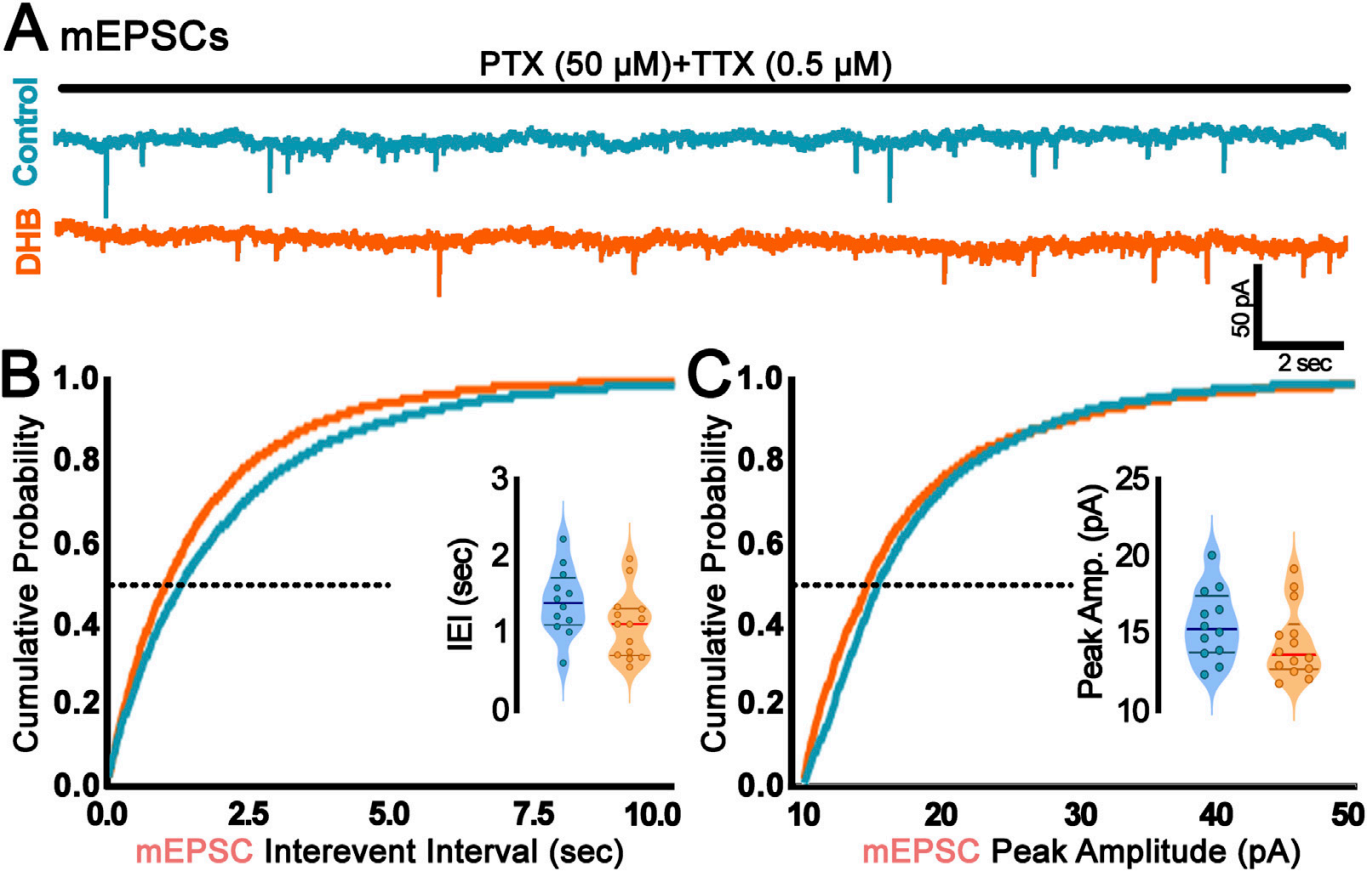
Incubation with 3-β-hydroxybutyrate glycerides (DHB) did not change probabilistic quantal release of glutamatergic vesicles. **(A)** Representative traces of miniature Excitatory Postsynaptic Currents (mEPSC) recorded from control (blue) or DHB (orange) CA1 pyramidal neurons. Scale bar: 50 pA and 2 s. **(B)** Cumulative probability distribution of mEPSC interevent interval (sec); inset: average mEPSC interevent interval at P50 (Control: 1.41 ± 0.12; 12 cells, 4 animals vs. DHB: 1.11 ± 0.11; 14 cells, 5 animals; unpaired t-test, p = 0.0794). **(C)** Cumulative probability distribution of mEPSC peak amplitude (pA); inset: average mEPSC peak amplitude at P50 (Control: 15.55 ± 0.66; 12 cells, 4 animals vs. DHB: 14.38 ± 0.62; 14 cells, 5 animals; unpaired t-test, p = 0.2071).

Since we found DHB-mediated alterations in sEPSCs, a further investigation was conducted to verify whether such changes could be attributed to synaptic transmission rather than spontaneous presynaptic action potentials. This was achieved through the recording of miniature spontaneous excitatory postsynaptic currents (mEPSCs), which provide valuable insights into the probabilistic quantal release of glutamatergic (excitatory) vesicles and the function of AMPA receptors. Figure 3 presents the results of the mEPSC analysis, revealing no statistically significant difference between the conditions of our experimental treatment with DHB (inter-event interval: control: 1.41 ± 0.12 vs. DHB: 1.11 ± 0.11; p = 0.08; peak amplitude: control: 15.55 ± 0.66 vs. DHB: 14.38 ± 0.62; p = 0.21). These results suggest that, within the parameters assessed, DHB did not produce detectable modifications in the quantal excitatory synaptic transmission.

### 3.3 CA1 neurons incubated with 3-β-hydroxybutyrate glycerides exhibit increased saturation of postsynaptic response

**FIGURE 4:**
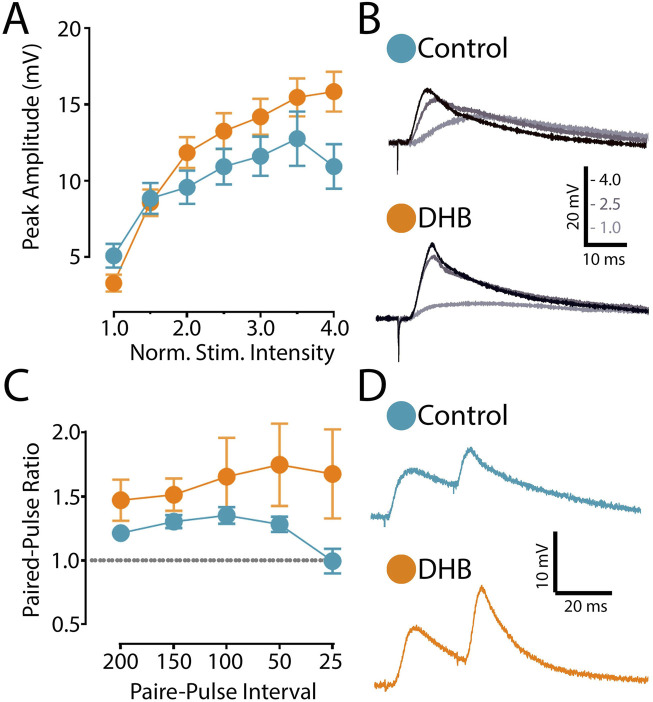
Incubation with 3-β-hydroxybutyrate glycerides (DHB) increases the saturation of excitatory postsynaptic responses. **(A)** Evoked excitatory postsynaptic potential (eEPSP) magnitude (mV) recorded from CA1 pyramidal neurons from Control (14 cells, 9 animals) and from slices exposed to 2 h DHB (2 mM) (10 cells, 4 animals). The stimulation intensities are normalized with respect to the intensity value evocating the minimal response. Two-Way ANOVA with Bonferroni posttest: DHB p = 0.2257; Stimulus Intensity p < 0.0001, Interaction p < 0.0001. **(B)** For each group, example traces of recorded eEPSP for 1.0, 2.5 and 4.0 normalized intensities are shown. **(C)** Paired-pulse ratio of eEPSP with different inter-stimulus interval (ms): five different interval times between stimulation were evaluated (200 ms, 150 ms, 100 ms, 50 ms, and 25 ms). Plotted data are the average ratio from second to first pulse (Control: 12 cells, 8 animals; DHB: 13 cells, 7 animals). Two-Way ANOVA with Bonferroni post test: DHB p = 0.1532; Pulse interval p = 0.2206, Interaction p = 0.1008. **(D)** Representative eEPSP traces from Pyramidal CA1 show the results of an experiment with the average of paired-pulse postsynaptic responses recorded with 25 ms inter-stimulus-interval of Control and 2 h DHB exposed neuron.

We then investigated the possibility that DHB treatment modulates the main synaptic input to CA1 pyramidal neurons. The stimulation electrode was positioned upon presynaptic projections coming from the CA3 region. In contrast, pyramidal neurons in the CA1 region were recorded in current clamp mode (Figure 4A). Evoked excitatory postsynaptic potentials (eEPSP) were evoked by applying a pulse of current (pA).

The synaptic strength of this connection was assessed by generating an input-output curve, where we recorded the postsynaptic responses to stimuli of increasing intensity. In these experiments, we compared cells recorded in the control condition with cells previously incubated with DHB. After obtaining the eEPSP elicited by the minimal stimulation, we recorded postsynaptic responses caused by increased stimulation intensities. As shown in Figures 4 A,B, our data indicates that neurons treated with DHB reach higher postsynaptic amplitudes at saturation levels (p < 0.0001; two-way ANOVA). This result demonstrates that CA1 hippocampal neurons treated with DHB can recruit a greater number of synaptic inputs in response to increased stimulation intensities

Next, to investigate the possibility that DHB might modulate the presynaptic mechanism of neurotransmitter release, we used the paired-pulse ratio (PPR) protocol, which induces a form of short-term synaptic plasticity. We examined the PPR with different time intervals between pulses and compared DHB-treated neurons with control cells. Our analysis revealed no significant differences (Figures 4 C,D), suggesting that the modulatory effect of DHB on synaptic transmission does not involve changes at the presynaptic terminals (p = 0.1008; two-way ANOVA).

### 3.4 Treatment with 3-β-hydroxybutyrate glycerides reduces long-term potentiation in the CA1 region

We investigated the possibility that DHB might play a metaplastic role and modulate synaptic plasticity induced by high-frequency stimulation (HFS). In these experiments, we adopted the extracellular recording of field potentials on the CA3-CA1 synaptic pathway.

**FIGURE 5:**
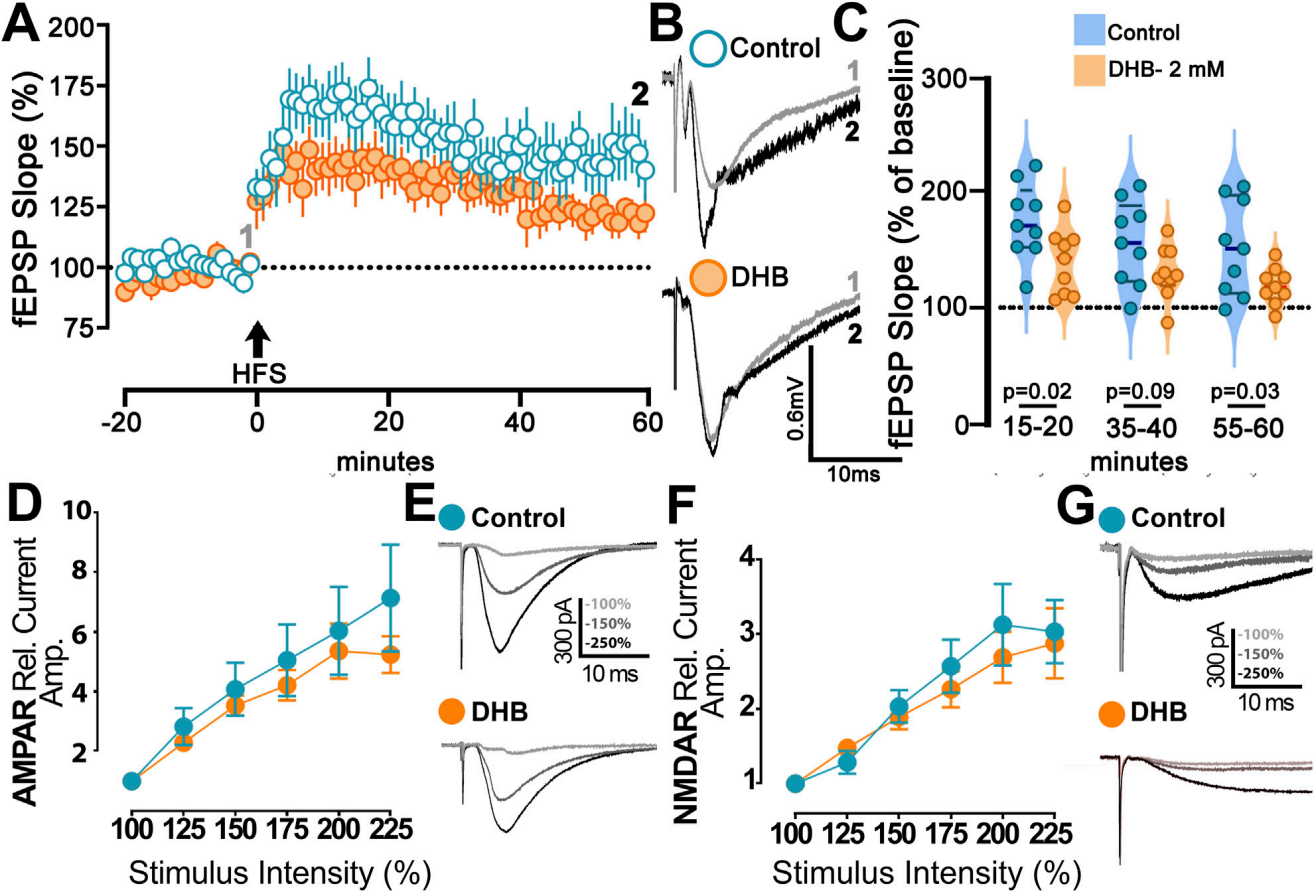
B-hydroxybutyrate effects on synaptic plasticity. **(A)** Long-term Potentiation (LTP) at CA1 induced by high frequency stimulation (HFS; 4 × 100 Hz) of Control (blue) and DHB slices (orange) (Control: 10 slices, 9 animals; DHB: 9 slices, 8 animals). DHB slices were 2 h treated with DHB (2 mM) before recordings. Time-course of field excitatory postsynaptic potential (fEPSP; slope), percentual change from baseline, plotted data are average ± SEM (Two-Way repeated measures ANOVA with Bonferroni posttest: DHB p = 0.0411; HFS response p < 0.0001, Interaction p = 0.0005). **(B)** Examples of Control and DHB fEPSP traces showing the magnitude of change from baseline (gray) and 60 min after HFS (black). **(C)** Violin plot of percentual field-EPSP (slope) after HFS at 15–20 min (176.5 ± 10.9 vs. 141.2 ± 9.4; unpaired t-test, p = 0.0262), 35–40 min (157.7 ± 12.2 vs. 132.0 ± 7.7; unpaired t-test, p = 0.0924) and 55–60 min (153.2 ± 13.7 vs. 120.3 ± 5.4; unpaired t-test, p = 0.0397) after HFS. **(D)** Magnitude of AMPAr excitatory postsynaptic current (EPSC) recorded from Control and slices exposed to 2 h DHB (2 mM). Plotted data are average from percentual change from minimal stimulation intensity (Control: 6 cells, 2 animals; DHB: 5 cells, 3 animals). The stimulation intensities are normalized with respect to the intensity value evocating the minimal response. Analysis showed no difference between Control and DHB group (Two-Way repeated measures ANOVA with Bonferroni posttest: DHB p = 0.6287; Stimulus intensity p < 0.0001, Interaction p = 0.8706). **(E)** Examples of AMPAr-EPSCs traces of Control and DHB group showing the amplitude with higher intensities of stimulation 100% (gray), 150% (dark gray) and 225% (black). **(F)** Magnitude of NMDAr excitatory postsynaptic current (EPSC) recorded from Control and slices exposed to 2 h DHB (2 mM). Plotted data are average from percentual change from minimal stimulation intensity (Control: 10 cells, 6 animals; DHB: 13 cells, 6 animals). The stimulation intensities are normalized with respect to the intensity value evocating the minimal response. Analysis showed no difference between Control and DHB group (Two-Way repeated measures ANOVA with Bonferroni post test: DHB p = 0.5477; Stimulus intensity p < 0.0001, Interaction p = 0.9446). **(G)** Examples of NMDAr-EPSCs traces of Control and DHB group showing the amplitude with higher intensities of stimulation 100% (gray), 150% (dark gray) and 225% (black).

Our results showed that HFS caused an increase in postsynaptic response in control CA1 neurons and in cells treated with DHB (p = 0.031; Two-Way ANOVA) (Figures 5A–C). Figure 5C shows the field excitatory postsynaptic potential (fEPSP) changes caused by the HFS protocol for the two groups. We made a comparison for the long-term potentiation (LTP) between the DHB group and control cells at three different periods, and we found that tissues treated with the compound exhibited a lower fEPSP slope when compared to the control group (15–20 min; 176.5 ± 10.9 vs. 141.2 ± 9.4; p = 0.026; 35–40 min, 157.7 ± 12.2 vs. 132.0 ± 7.7, p = 0.092; 55–60 min, 153.2 ± 13.7 vs. 120.3 ± 5.4; p = 0.0397; Control: 10 slices, 9 animals; DHB: 9 slices, 8 animals).

We then studied the activities of AMPA and NMDA receptors to evaluate their role in DHB-mediated plasticity and metaplasticity. In these experiments, we compared cells incubated for 2 hours with DHB with control cells. Isolation of AMPA or NMDA receptor currents was achieved through the application of specific blockers, as described in the methods. Deliverance of progressively higher current intensities generated postsynaptic response with a gradual increase in amplitude. However, no significance between the DHB and Control group was found for the responses mediated by AMPA receptors (Figures 5D,E; p = 0.96; Two-Way ANOVA), even by NMDA receptors (Figures 5F,G; p = 0.92; Two-Way ANOVA). This finding indicates that synaptic transmission from the CA3 to CA1 input, mediated by AMPA and NMDA receptors remained stable across experimental conditions, suggesting that DHB did not exert measurable effects on this specific aspect of excitatory neurotransmission.

### 3.5 The DHB-mediated effects do not involve the activation of HCAR2

Although DHB is known to modulate brain function through the activation of Hydroxycarboxylic Acid Receptor 2 (HCAR2), several distinct pathways might contribute to its intracellular signalling (Wang et al., 2023). Consequently, we aimed to investigate if the observed effects in our electrophysiology experiments were explicitly linked to the activation of HCAR2. To address this possibility, brain slices were pre-incubated for 2 hours with niacin (an HCAR2 agonist) before recording.

**FIGURE 6:**
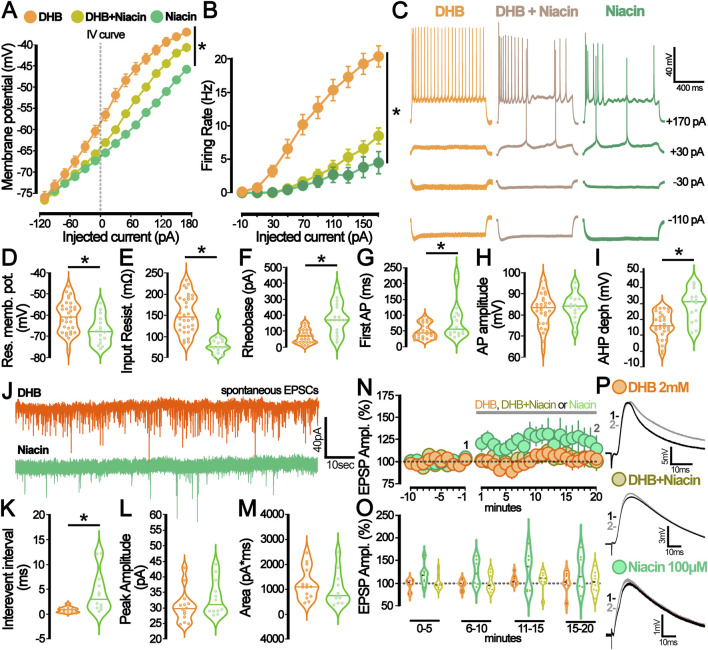
The hydroxycarboxylic acid receptor 2 (HCAR2) activation by niacin diverges from the β-hydroxybutyrate (DHB) effect on neuronal excitability and synaptic modulation. **(A)** Current-Voltage plot (I-V curve) for the whole-cell current measurements (DHB: 24 cells, 14 animals (orange); niacin + DHB: 24 cells, 7 animals (yellow), niacin: 16 cells, 7 animals (green), p < 0.0001 for all comparisons, Two-Way ANOVA. **(B)** Graph representing the number of action potentials deflagrated by current injection (DHB: 20 cells, 14 animals (orange); niacin + DHB: 24 cells, 7 animals (yellow), niacin: 16 cells, 7 animals (green); p < 0.0001 for all comparisons, Two-Way ANOVA. **(C)** Representative traces obtained from CA1 pyramidal neurons submitted to the current injection protocol under the prolonged incubation with DHB 2 mM (orange), DHB 2 mM + niacin 100 μM (yellow), and niacin 100 μM (green). **(D)** Graph of the resting membrane potential (DHB: −60.6 ± 1.3 mV; 31 cells, 7 animals (orange) vs. niacin: −67.1 ± 1.6 mV; 16 cells, 7 animals (green); p = 0.0049 in unpaired t-test). **(E)** Graph of the Input resistance (DHB: 150 ± 0.8 mΩ; 32 cells, 7 animals (orange) vs. niacin: 80 ± 0.6 mΩ; 16 cells, 7 animals (green); p < 0.0001 in unpaired t-test). **(F)** Graph of the Rheobase (DHB: 67.93 ± 8.21 pA; 29 cells, 7 animals (orange) vs. niacin:181.3 ± 24.42 pA; 16 cells, 7 animals (green) p < 0.0001 in Mann-Whitney test. **(G)** Graph of the time of the first action potential (DHB: 50.61 ± 4.41 ms; 29 cells, 7 animals (orange) vs. niacin: 84.06 ± 15.28 ms; 16 cells, 7 animals (green), p = 0.0348 in Mann-Whitney test). **(H)** Graph of the action potential (AP) amplitude (DHB: 82.41 ± 1.47 mV, 29 cells, 7 animals (orange) vs. niacin: 84.23 ± 1.65 mV; 14 cells, 7 animals (green), p = 0.46 in unpaired t-test). **(I)** Graph of the after-hyperpolarization potential (AHP) depth (DHB: 16.28 ± 1.5 mV, 29 cells, 7 animals (orange) vs. Niacin: 28.53 ± 2.74 mV, 14 cells, 7 animals (green), p = 0.0001 in unpaired t-test). **(J)** Representative traces from spontaneous excitatory postsynaptic currents (sEPSC) recorded from CA1 pyramidal neurons incubated with DHB 2 mM (orange) and niacin 100 μM (green). **(K)** Graph of the interevent interval of the sEPSC at P50 (DHB: 0.89 ± 0.15 ms; 13 cells, 7 animals (orange) vs. niacin: 4.46 ± 1.19; 12 cells, 7 animals (green); p = 0.0051 in Mann-Whitney test). **(L)** Graph of the peak amplitude of the sEPSC at P50 (DHB: 30.5 ± 1.38; 14 cells, 7 animals (orange) vs. niacin: 33.25 ± 1.63; 12 cells, 7 animals (green); p = 0.25 in Mann-Whitney test). **(M)** Graph of the area of the sEPSC at P50 (DHB: 1231 ± 168.6; 14 cells, 7 animals (orange) vs. niacin: 1014 ± 203.2; 11 cells, 7 animals (green); p = 0.41 in unpaired t-test). **(N)** Graph of the relative amplitude of the evoked excitatory postsynaptic potential (eEPSP) recorded from CA1 pyramidal neurons before and during DHB (2 mM, orange), DHB + niacin (2 mM and 100 μM, yellow), and niacin (100 μM, green) bath application (grey bar). **(O)** Summary of the postsynaptic response after DHB (orange), DHB + niacin (yellow), and niacin (green) bath application. The violin plot shows the average of eEPSP values recorded during different periods of the experiment: 0–5 min, 6–10 min, 11–15 min, and 15–20 min. **(P)** Representative average trace from the baseline (1-black,-5 to −1 min) and after bath application (2- grey, 15–20 min) of DHB (2 mM, orange), DHB + niacin (2 mM and 100 μM, yellow), and niacin (100 μM, green).

To investigate the individual effects of niacin or its combination with DHB, we conducted electrophysiological assays similar to those previously employed. Analysis of membrane potential (Figure 6A) provided detailed insight into the hyperpolarizing effects of niacin on pyramidal CA1 neurons. The data demonstrated that niacin induces a significant hyperpolarization of the neuronal resting membrane potential. However, this effect was less pronounced when cells had undergone pre-treatment with DHB. This attenuation suggests that while both niacin and DHB impact membrane potential, their mechanisms of action are distinct and independent. Figure 6B presents the analysis of the number of action potentials per injected current. In the group where brain slices were pre-incubated with niacin, 11 out of 16 recorded cells did not generate action potentials in response to a −170 pA current injection (1000 ms). In contrast, in the group that received both niacin and DHB treatment, only 5 out of 24 recorded cells failed to generate action potentials under the same conditions. Both groups that received niacin exhibited a reduction in the number of action potentials compared to the Control or DHB groups (p < 0.0001, two-way ANOVA) - Figure 6C shows representative traces. A more detailed analysis of passive and active membrane properties (Figures 6D–I) revealed that the previously observed effects of DHB were independent of HCAR2 receptor activation. This conclusion is supported by the fact that, for the majority of evaluated parameters, the responses of cells treated with DHB differed significantly from those only submitted to HCAR2 receptor activation through the use of its agonist, niacin (Resting membrane potential: p = 0.0049; Input resistance: p < 0.0001; Rheobase: p < 0.0001; Time to first spike: p = 0.0348; After hyperpolarization depth: p = 0.0002). The analysis of spontaneous excitatory postsynaptic currents (sEPSC) (Figures 6J–M) revealed that only treatment with DHB was effective in reducing the inter-event interval. These findings suggest that DHB exerts its effects mostly through mechanisms distinct from those mediated by HCAR2 activation, reinforcing the idea that DHB and niacin engage separate physiological pathways.

To explore whether such modifications would already be detectable in acute assays, we investigated the immediate effect on evoked excitatory postsynaptic potential (eEPSP from CA3-CA1) when the neuron was exposed to a solution containing DHB (2 mM), niacin (100 mM), or a combination of both (DHB and Niacin) (Figures 6N–P). The absence of significant differences in evoked currents among the experimental groups (Figure 6N; p = 0.3436, two-way ANOVA) suggests that the immediate exposure to DHB, niacin, or their combination does not induce acute bioelectrical modifications detectable in this assay. This finding highlights that DHB’s physiological effects on neural activity mostly rely on chronic and long-term intracellular processes.

## 4 Discussion

This study aimed to investigate the effects of 3-β-hydroxybutyrate glycerides (DHB) on the intrinsic properties of hippocampal CA1 neurons and synaptic activity. The two main findings of this work revealed that DHB enhances the excitability of hippocampal neurons and that this response does not occur exclusively through the activation of its membrane receptor.

We found that depolarizing steps of currents applied to the recorded CA1 pyramidal neurons in cells previously incubated with DHB exhibit a higher frequency of action potential firing (increased intrinsic excitability) with preserved synaptic inputs. Additionally, DHB raises the density of spontaneous Excitatory Postsynaptic Currents (sEPSC) while maintaining GABAergic activity unchanged. The DHB-mediated effect on sEPSC may be produced by enhanced presynaptic efficiency or by a higher frequency of spontaneous action potentials in presynaptic neurons. We propose that the latter scenario is more plausible, as our study on the paired-pulse ratio (PPR) demonstrated that DHB did not increase the presynaptic efficacy of the CA3-CA1 synapse. Furthermore, the DHB-mediated increase of event frequency has not been found in the recording of miniature Excitatory Postsynaptic Currents (mEPSC). Therefore, the effect of DHB on spontaneous excitatory activity is attributable to the enhanced excitability of the presynaptic cells, which, in turn, leads to an increase in postsynaptic spontaneous events. Our results also indicated that the postsynaptic response evoked through Schaffer collateral stimulation attains a higher saturation plateau in cells previously incubated with DHB. This effect can be attributed to an increase in the excitability of CA3 cells and may reflect the recruitment of a larger number of fibers with increasing stimulation intensity. Thus, DHB appears to cause increased excitability in both presynaptic afferences from CA3 and postsynaptic neurons of CA1. Experiments aimed to study synaptic plasticity showed that incubation with DHB reduces the long-term potentiation (LTP) at CA3 to CA1 connections. Since no changes were observed in NMDA and AMPA receptor currents, the modulation of LTP does not appear to result from decreased calcium influx into the postsynaptic neuron. Instead, it may be linked to downstream alterations in intracellular signaling. We then explored the possibility that these effects are primarily mediated through activation of the membrane receptor Hydroxycarboxylic Acid Receptor 2 (HCAR2); however, we observed opposing responses when hippocampal pyramidal neurons were treated solely with its agonist. Extracellular activation of this metabotropic receptor led to a reduction in neuronal excitability (in both evoked and spontaneous activity), thereby indicating that intracellular DHB metabolism is necessary to mediate its overall effect.

Although it is widely recognized that hydrolysis by pancreatic esterases contributes to its metabolism, evidence suggests that DHB is locally metabolized into free β-hydroxybutyrate (BHB) through the action of esterase enzymes present in brain tissue cells (Jones et al., 2013; Singh et al., 2021). The two major cell types primarily involved in the metabolism of ketone esters are neurons and astrocytes. Both express esterases capable of cleaving the ester bond in compounds such as DHB, thereby releasing free BHB into the extracellular or intracellular microenvironment. Recent findings have demonstrated that neurons, which are highly energy-dependent and metabolically active, both in KD and in hyperglycemia, BHB is used as the main substrate for ATP production, protecting the brain by improving mitochondrial efficiency (García-Rodríguez and Giménez-Cassina, 2021; Holstein et al., 2025). Consequently, intracellularly generated BHB is rapidly consumed through the tricarboxylic acid (TCA) cycle for energy synthesis, and BHB released into the extracellular microenvironment of the brain slice may be swiftly taken up by neighboring neurons and similarly directed toward mitochondrial ATP generation.

The existing literature presents a complex and contradictory scenario regarding the effects of ketone bodies (KBs) and ketogenic diets (KDs) on synaptic plasticity. While there is a consensus that these compounds enhance cognitive abilities, their direct impact on synaptic plasticity is context-dependent. For instance, a previous study by Qiao et al. (2024) evaluated the effects of KD on cognitive functions in an epilepsy model. They found that KD reduces the endoplasmic reticulum stress (ERS) and improves synaptic plasticity in the hippocampus. They demonstrated that KD improved learning and memory, suppressed excessive ERS induced by epilepsy, enhanced the density of dendritic spines, and reversed the LTP deficit induced by epilepsy. In contrast, another work by Koranda et al. (2011) showed a decrease in the LTP in the dentate gyrus of rodents after 3 weeks on a KD (Koranda et al., 2011). The cognitive benefits often associated with KDs in humans (Halyburton et al., 2007; Brinkworth, 2009) these cognitive benefits might not always be directly mediated by increased LTP in hippocampal circuitry. Further evidence from Qiao et al. (2024) suggests that prolonged exposure (28 days) to KBs in the brain can lead to improvements at the protein level, resulting in enhanced synaptic plasticity and cognitive function. They found that KD was accompanied by increased expression of postsynaptic density protein 95, synaptotagmin-1, and synaptosomal-associated protein 25 in the hippocampi - all proteins directly linked to improved synaptic plasticity and cognitive function. It suggests that the duration of exposure to KBs may be a crucial factor in determining their effects on synaptic plasticity.

Unlike previous studies examining the effects of a KD on neuroplasticity - an approach that may involve multiple mediating processes - our research identifies a direct effect of a ketogenic compound on neuronal cells, specifically inhibiting LTP. We hypothesize that DHB may act by influencing homeostatic plasticity mechanisms. Given that DHB enhances both excitability and synaptic efficacy, it is plausible that it may simultaneously induce a reduction in potentiation levels to prevent hyperexcitation of the neural circuit, thereby maintaining synaptic transmission within a physiological range.

The Energy Homeostasis Principle, as proposed by Vergara et al. (2019), posits that neuronal dynamics across molecular, cellular, and behavioral scales are fundamentally governed by the balance between energy input, expenditure, and availability. A substantial portion of neural energy consumption is attributed to the generation of action potentials and postsynaptic potentials. Despite the tight coupling between activity and energy use, the prediction of synaptic plasticity outcomes remains complex and cannot be solely inferred from neural firing patterns. On a biochemical level, β-hydroxybutyrate (BHB) serves as an alternative metabolic substrate, converted into acetyl-CoA and subsequently entering the tricarboxylic acid (TCA) cycle. This process yields NADH and FADH_2_, which drive ATP production within the inner mitochondrial membrane (Veech et al., 2001; Vidali et al., 2015). Exposure to DHB may enhance ATP availability, as neurons exhibit pronounced sensitivity to energy constraints, and free energy dictate the directionality of cellular metabolic processes, the increase in ATP may underlie the enhanced excitability observed in hippocampal neurons.

Considering that we worked with healthy subjects, we may suggest that the increase in excitability leads to dynamic compensatory adjustment, termed “homeostatic plasticity” - a fundamental regulatory function by which the brain normalizes its excitability (Turrigiano, 2008). In this context, it is reasonable that a more excitable tissue would exhibit a reduction in LTP, which occurs within a homeostatic framework and serves to prevent the emergence of pathological hyperexcitability.

Previous experiments conducted in GABAergic neurons of the substantia nigra pars reticulata and dentate granule cells of the hippocampus have demonstrated that K_ATP_ channels are indirectly activated in the presence of acetoacetate (2 mM) or β-hydroxybutyrate (2 mM), resulting in reduced neuronal excitability (Ma et al., 2007; Tanner et al., 2011). In this context, the observed reduction in the activity of GABAergic neurons, which exert tonic inhibition over CA1 pyramidal neurons, may be associated with the increased action potential frequency recorded in CA1 pyramidal neurons in the present study. This suggests a possible direct effect of BHB on these GABAergic neurons, with consequential modulation of pyramidal neuron excitability. Supporting this interpretation, previous studies have highlighted the critical role of tonic inhibition mediated by GABA_A_R in CA1 pyramidal neurons, which not only contributes to pathological hyperexcitability states, such as those observed in chronic epilepsy models, but also plays a key role in memory regulation (Caraiscos et al., 2004).

Clinical studies indicate that KD diminishes neuronal hyperexcitability manifested in neuronal disorders such as migraine, epilepsy, Parkinson’s disease, and Alzheimer’s disease (Simeone and Simeone, 2024; Hertz et al., 2015; Gross et al., 2019; Cheng et al., 2020; Fila et al., 2023; Iyer et al., 2024). Beyond its metabolic function, BHB also acts as a signaling molecule by targeting the G-protein-coupled membrane receptor HCAR2, which is broadly expressed across the central nervous system and implicated in long-term neurological effects (Shenol et al., 2024). BHB can reach neurons through specific proteins in the blood-brain barrier (MCT1 and MCT2) (Pellerin et al., 2005), and the HCAR2-Gi1 protein complex, is activated by BHB, triggers the cellular signaling pathway associated with the Gi/o protein (Mao et al., 2023). Our findings indicate that HCAR2, via niacin activation, has effects that are opposite to those observed with the full action of BHB (DHB excitability), which include neuronal hyperpolarization and an increased rheobase in healthy mice. This selective engagement of the membrane-bound receptor may play a crucial role in modulating neuronal excitability. This modulation, in turn, may play a crucial role in the excitability reduction frequently reported in studies linking the neuroprotective properties of the KD to a spectrum of brain disorders characterized by excitatory/inhibitory (E/I) imbalance. By directly influencing neuronal firing through this receptor, KBs could contribute to restoring a more balanced neuronal network, thereby alleviating symptoms and promoting neural health in these conditions.

In addition to overexcitability, aging is unquestionably accompanied by memory loss. Newman et al. (2017) studied the effects of KD throughout aging (12–24 months of age) in C57Bl-6 mice; their evaluations included a diverse set of cognitive and physical function tasks and demonstrated that KD group performed markedly better than the Control group in the memory/recall portion, a memory improvement accompanied by amelioration of age-related decline in physical performance. Our findings suggest that BHB modulates neuronal excitability in part through activation of its membrane receptor, HCAR2. This receptor-mediated signaling appears to play a key role in dampening both evoked and spontaneous activity, which may represent an early mechanism for counteracting the hyperexcitability commonly observed with ageing. Instead, a coordinated interplay between membrane signaling and intracellular actions of BHB seems to be necessary to support long-term synaptic plasticity, as reflected in sustained LTP performance. An example of this can be drawn from the study that addressed a highly prevalent condition in the elderly population, the Alzheimer’s disease: the group of Di Lucente et al. (2024) presented important results on synaptic plasticity in APP/PS1 models, where both KD and BHB were able to increase LTP values recorded in hippocampal slices after high-frequency stimulation (HFS). Note that once again, the effect of BHB was compared against a pathological state that presents damage in healthy neuronal circuitry. Thus, it is presented as a beneficial effect (since it is close to the response of a “normal” individual).

As an important survival mechanism, in conditions where BHB levels are elevated and glucose availability is reduced - such as during prolonged fasting - BHB acts as a neuroprotective agent, promoting adaptive mechanisms to preserve hippocampal function without compromising its essential operations. Under these metabolic constraints, the brain operates in an energy-conserving mode, optimizing its functional efficiency. A critical aspect of this adaptation is the intricate interplay between brain energy homeostasis and glutamatergic metabolism. Disruptions in glutamate clearance are known to induce neuronal overstimulation and excitotoxicity, contributing to the pathophysiology of various neurodegenerative disorders (Sepkuty et al., 2002). Among neural signaling pathways, excitatory glutamatergic neurotransmission demands the highest energy expenditure in the brain (Harris et al., 2012; Yu et al., 2018). Our findings reveal a notable homeostatic regulation: even under increased excitability induced by BHB exposure in the presence of optimal glucose levels, there is no corresponding elevation in glutamate release or signaling. As BHB is directly metabolized within mitochondria, its activity bypasses glycolysis and even suppresses it through enhanced mitochondrial metabolism, resulting in increased ATP production via oxidative phosphorylation and reduced ATP generation through glycolysis (Lutas and Yellen, 2013). This finding highlights the tight regulation of homeostatic mechanisms, which maintain adequate brain function within narrow physiological limits, even in the presence of increased energy supply. Such regulatory precision appears crucial for safeguarding neuronal integrity under varying metabolic states.

In conclusion, our results demonstrate that exposure to β-hydroxybutyrate glycerides is capable of triggering important functional changes. These modifications, which restrain a homeostatic range, have been observed in “healthy” individuals without cognitive loss or deficits associated with pathologies. The results provided by our work suggest that DHB may modulate hippocampal neural activity by increasing excitability and excitatory synaptic transmission while homeostatically downregulating LTP. Based on probabilistic reasoning, DHB is expected to interact with HCAR2 when this receptor is present. However, under physiological conditions, the relative contribution of HCAR2 activation appears to be limited when compared to the broader intracellular effects elicited by DHB. Collectively, these observations suggest that although HCAR2 plays a supporting role in the overall response (the activation of this receptor may be proportionally more relevant in pathological conditions), it does not fully explain DHB’s functional impact, which likely arises from a convergence of receptor-mediated and receptor-independent mechanisms.

## Data Availability

The raw data supporting the conclusions of this article will be made available by the authors, without undue reservation.

## Abbreviations

BHB, β-hydroxybutyrate; DHB, 3-β-hydroxybutyrate glycerides; AMPA, α-amino-3-hydroxy-5-methyl-4-isoxazolepropionic acid; NMDA, N-methyl-D-aspartate; LTP, long-term potentiation; HCAR2, Hydroxycarboxylic Acid Receptor 2; KD, ketogenic diet; KB, ketone bodies; aCSF, artificial cerebrospinal fluid; AP, action potential; eFEL, Electrophys Feature Extraction Library; AHP, after-hyperpolarization; sEPSC, spontaneous Excitatory Postsynaptic Currents; sIPSC, spontaneous Inhibitory Postsynaptic Currents; mEPSC, miniature spontaneous Excitatory Postsynaptic Currents; MSI, minimal stimulation intensity; eEPSP, evoked excitatory postsynaptic potential; PPR, paired-pulse ratio; fEPSP, field excitatory postsynaptic potential; HFS, high-frequency stimulation; AUC, area under the curve.
